# High Carotenoid Mutants of *Chlorella vulgaris* Show Enhanced Biomass Yield under High Irradiance

**DOI:** 10.3390/plants10050911

**Published:** 2021-05-01

**Authors:** Zeno Guardini, Luca Dall’Osto, Simone Barera, Mehrdad Jaberi, Stefano Cazzaniga, Nicola Vitulo, Roberto Bassi

**Affiliations:** Dipartimento Di Biotecnologie, Università Di Verona, Strada Le Grazie 15, 37134 Verona, Italy; zeno.guardini@univr.it (Z.G.); luca.dallosto@univr.it (L.D.); simone.barera@univr.it (S.B.); mehrdad.jaberi@univr.it (M.J.); stefano.cazzaniga@univr.it (S.C.); nicola.vitulo@univr.it (N.V.)

**Keywords:** microalgae, biomass, photoprotection, carotenoids, photooxidative stress, chloroplast, norflurazon

## Abstract

Microalgae represent a carbon-neutral source of bulk biomass, for extraction of high-value compounds and production of renewable fuels. Due to their high metabolic activity and reproduction rates, species of the genus *Chlorella* are highly productive when cultivated in photobioreactors. However, wild-type strains show biological limitations making algal bioproducts expensive compared to those extracted from other feedstocks. Such constraints include inhomogeneous light distribution due to high optical density of the culture, and photoinhibition of the surface-exposed cells. Thus, the domestication of algal strains for industry makes it increasingly important to select traits aimed at enhancing light-use efficiency while withstanding excess light stress. Carotenoids have a crucial role in protecting against photooxidative damage and, thus, represent a promising target for algal domestication. We applied chemical mutagenesis to *Chlorella vulgaris* and selected for enhanced tolerance to the carotenoid biosynthesis inhibitor norflurazon. The *NFR* (norflurazon-resistant) strains showed an increased carotenoid pool size and enhanced tolerance towards photooxidative stress. Growth under excess light revealed an improved carbon assimilation rate of *NFR* strains with respect to WT. We conclude that domestication of *Chlorella vulgaris,* by optimizing both carotenoid/chlorophyll ratio and resistance to photooxidative stress, boosted light-to-biomass conversion efficiency under high light conditions typical of photobioreactors. Comparison with strains previously reported for enhanced tolerance to singlet oxygen, reveals that ROS resistance in *Chlorella* is promoted by at least two independent mechanisms, only one of which is carotenoid-dependent.

## 1. Introduction

Mass culture of microalgae in photobioreactors (PBRs) has gained interest in the past few decades. Beside small-scale traditional cultivations mainly aimed to human or animal feeding, commercial production of algae on a large scale has been identified as a renewable and environmentally sustainable strategy for feedstock production [1]. Both microalgae and land plants catalyze photosynthetic reactions [2], yet the simpler structure of microalgae enhances efficiency in solar energy conversion into biomass respect to plants [3,4,5,6]. Thus, cultivation of microalgae represents a promising source of biomass for many industrial applications, which include production of bioactive compounds, recombinant proteins, livestock feed, biofuels, organic fertilizer and biostimulants [7,8]. Microalgae can grow in wastewater on marginal lands, thus avoiding competition for both arable land and water source with food crops [9]. Furthermore, the high capacity for nitrogen and phosphorus removal, together with use of flue gas as a source of CO_2_, makes phycoremediation a valuable circular-economy-based biorefinery [10,11].

The genus *Chlorella* includes fast-growing species, highly productive and easy to cultivate, exploited to a significant extent as food additives and nutraceuticals [12]. Carotenoids (Car) are currently the most important commercial product from microalgae successfully marketed [13,14,15]. These pigments are widely utilized as aquaculture feed additives, food colorants and ingredients for cosmetics; moreover, Car have biomedical applications, as anti-inflammatory agents due to their strong antioxidant properties [1]. Xanthophylls (oxygenated Car) are antioxidants with a key role in protecting from ocular oxidative damage [16]; indeed, the human macula lutea are rich in lutein and zeaxanthin, which help maintain eye health. β-carotene is commercially produced mainly from the halophilic alga *Dunaliella salina* through cultivation in ponds; however, microalgae are not yet used as a source of xanthophylls at an industrial scale [7]. The latter and many other algae-based production processes require significant improvements in the high cost for PBRs management and in the efficiency at various process steps such as biomass recovery and product extraction [17]. Moreover, limitations include biological constraints such as low efficiency of PAR (photosynthetically active radiation) utilization, especially under excess light (EL) conditions, which hinder the economic viability of algal products. The maximum theoretical efficiency of solar energy conversion into biomass has been reported to fall between 6% and 12% [3,18] while present-day production systems typically exhibit photosynthetic conversion efficiency of about 1% or below [18,19]. Limits in biomass yield can be ascribed to a number of factors [20,21], including inhomogeneous light distribution within a mass culture and photooxidative stress.

In particular, PAR efficiently drives photosynthesis under light-limiting conditions, while photoinhibition is observed upon exposure to irradiance exceeding plant capacity for electron transport, decreasing light-to-biomass conversion efficiency [22]. The high optical density of wild-type strains accounts for additional efficiency drop: a high chlorophylls (Chl) content per cell is a valuable trait which maximizes the photon capture in the natural environment, while a large array of antenna complexes (light-harvesting complexes, LHCs) [23], binding Chl and Car [24], hampers biomass productivity in a PBR. Indeed, high cell concentration originates a steep light gradient which leaves inner cell layers below the compensation point [25], while sustained over-excitation of cells in the surface layers increases the yield of Chl triplet state (^3^Chl*) and the consequent release of the reactive oxygen species (ROS) singlet oxygen (^1^O_2_). Photooxidative stress is a consequence of the formation of strongly oxidizing intermediates in different steps of the photosynthetic process, that inevitably leads to PSII photoinhibition and reduces net assimilation [26,27].

Domesticating microalgae for higher productivity in mass cultures requires introduction of traits aimed to relieve the light-use constraints [28]. Among many, we chose two traits: (i) the optical density of algal cells and (ii) the photoprotection capacity. Decreasing overall absorption of PAR per unit volume of culture was shown to enhance light distribution in PBR and thus productivity [29]. Whereas, increasing resistance to photooxidative stress is expected to prevent photoinhibition, thus increasing fitness [30] and enhancing carbon gain [31].

In the photosynthetic membranes, the photoprotective action of Car is well-established [32,33]. Car participate in the antioxidant network of the chloroplast, aimed at the detoxification of ROS generated by light reactions: Car efficiently act by scavenging both O_2_^−^ and OH∙, and ^1^O_2_ in thylakoids, thus preventing lipid peroxidation. Chl-binding complexes are protected against ^1^O_2_ formation by Car, which catalyze quenching of ^3^Chl* and results in Car triplets (^3^Car*) formation, that safely decay to the ground state, producing heat [34,35]. Car biosynthesis is therefore a promising target to improve tolerance to photooxidative stress.

In this work, we report on the isolation of *Chlorella vulgaris* mutant strains combining lower optical density and higher resistance to EL stress. Through chemical mutagenesis followed by selection on the carotenoid biosynthesis inhibitor norflurazon, we succeeded in the isolation of two pale green strains enriched in the antioxidant Car (*NFR,* norflurazon-resistant strains). Mutants exhibited higher biomass productivity in PBR, and were significantly more resistant to photooxidative damage than WT strain under very strong irradiance. Moreover, *NFR* strains in PBR showed further enhancement in productivity with respect to another *Chlorella* pale green mutant [36].

Overall, these results demonstrate that domestication of *C. vulgaris* for improved tolerance to photooxidation has the potential to generate strains with positive advantages in the artificial environment of a PBR. Moreover, comparison with strains previously obtained [36], which were selected for resistance to ^1^O_2_, reveals that photoprotection in *Chlorella* is promoted by at least two independent mechanisms, only one of which is strengthened by Car overaccumulation.

## 2. Results

### 2.1. Isolation of NFR-3 and NFR-13, High Car Content Mutants of Chlorella vulgaris

Tolerance to high light stress is a desired trait for microalgal biotechnology [37], being crucial for establishing efficient outdoor cultivation in low latitudes. Car biosynthesis represents an obvious target when aiming to enhance photosynthetic yield of microalgae under photooxidative conditions caused by EL. Indeed, Car contribute to the antioxidant network of the chloroplast, aimed to the detoxification of ROS, particularly ^1^O_2_ generated by photosynthetic light reactions, thus preventing lipid peroxidation [38], whereas the photoacclimation to high light involves Car up-regulation [39]. We used random mutagenesis and phenotypic screening to isolate *C. vulgaris* strains that combined a pale green phenotype with increased size of Car pool, these being traits expected to improve light conversion efficiency. Cells were subjected to two rounds of random chemical mutagenesis with EMS and mutants were selected for resistance to norflurazon, an inhibitor of carotenogenesis [40]. In a first selection step, approximately 5000 mutagenized lines were screened and seven independent mutants were distinguished as putatively affected in Chl content per cell. Pale green strains were pooled, grown to enhance cell number, and submitted to a second treatment with EMS. Approximately 60,000 mutagenized lines were spread on BG-11 agar plates containing 4 μM norflurazon. 54 herbicide-resistant colonies (*NFR*, norflurazon-resistant) were obtained. Upon discarding mutants exhibiting either low growth or unstable resistance to the herbicide, 12 lines were selected, grown in liquid cultures, and the Car and Chl content per cell was determined after five days of growth under photoautotrophic conditions. Among the identified strains, lines *NFR-3* and *NFR-13* showed the highest Car content per cell, and were therefore selected for further analysis.

*NFR* lines showed a significant reduction of Chl content per cell (−58% in *NFR-3*, −73% in *NFR-13*) (Table 1). The Chl *a/b* ratio was significantly higher in the mutants, with a value of 4.14/4.92 in *NFR-3*/*-13* vs. 2.67 in WT. These data hint a reduction in the Chl *b* rich antenna complexes (LHC) in both mutants. Moreover, *NFR-13* strain showed a stronger decrease in total Chl per cell and a higher Chl a/*b* ratio, thus suggesting that the Chl *b*-enriched antenna system was further decreased in this line respect to *NFR-3*.

Both *NFR-3* and *NFR-13* exhibited higher Car content per cell (+25% and +14%, respectively) respect to the WT strain (Table 1). Additional HPLC analysis of the Car composition of cell extracts (Table 2, Figure S1) revealed that carotenes and xanthophylls, while more abundant in *NFR* on a Chl basis, were maintained in similar relative abundance compared to WT.

Further to the characterization of these mutants, we checked the effect of nitrogen starvation on Car accumulation, a treatment reported to increase Car content [41]. When transferred from standard BG-11 to a modified BG-11 medium with limiting N source (NaNO_3_ 0.8 mM), Car contents on a per cell basis increased in both *NFR* strains, by 1.7- and 2.5-fold, respectively, than standard BG-11, with the β,β-xanthophylls undergoing a preferential enhancement over α,β-xanthophylls and carotenes (Figures S1 and S2, Table 2).

### 2.2. Stoichiometry of Pigment–Protein Complexes and Photosynthetic Efficiency

**Figure 1:**
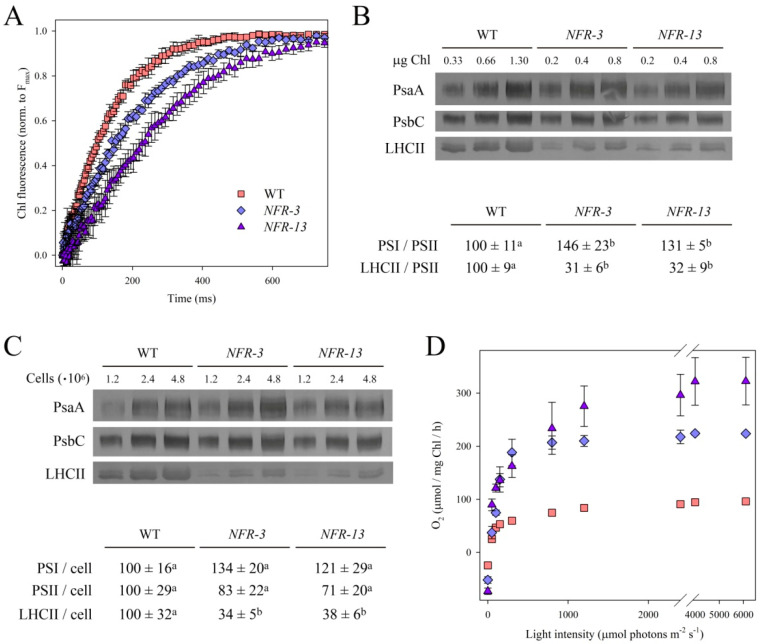
Characterization of *C. vulgaris norflurazon-resistant* (*NFR*) strains of *Chlorella vulgaris*. (**A**) PSII functional antenna size. Variable Chl fluorescence was induced with a green light (15 μmol photons m^−2^ s^−1^), on dark-adapted cells of WT and *NFR* (about 1 × 10^7^ cells mL^−1^) in BG-11 medium supplemented with 50 μM DCMU. Data are expressed as mean ± SD, *n* = 10. The reciprocal of time needed for reaching two-thirds of the fluorescence rise (T_2/3_) was taken as a measure of the PSII functional antenna size (see Table 1). (**B**,**C**) Immunoblotting used for the quantification of photosynthetic subunits. (*upper*) Immunotitration was performed with antibodies directed against individual gene products: LHCII, the major light harvesting complex of PSII; the PSII core subunit PsbC (CP43); the PSI core subunit (PsaA). The amounts of Chls (panel **B**) and of cells (panel **C**) loaded for each lane are indicated. (*lower*) In each table, significantly different values (ANOVA followed by Tukey’s post-hoc test at a significance level of *p* < 0.05), within the same row, are marked with different letters. (**D**) Light-saturation curves of photosynthesis, measured in cultures grown in BG-11 minimal medium. Data are expressed as mean ± SD, *n* = 4.

To determine whether the mutation affected the PSII operating efficiency, Chl fluorescence measurements were carried out to quantify the capacity of the antenna system to transfer absorbed energy to RCs. All strains showed no significant differences in the maximal quantum yield of PSII photochemistry (F_v_/F_m_) (Table 1), suggesting that the PSII function was not worsened in *NFR* mutants despite the altered pigment composition. PSII functional antenna size was evaluated upon treatment of cells with DCMU (3-(3,4-dichlorophenyl)-1,1-dimethylurea), an inhibitor that blocks the plastoquinone binding site of PSII. In DCMU-treated cells, the rise time of Chl *a* fluorescence (Figure 1A) is inversely related to the antenna cross-section [42,43]. The T_2/3_^−1^ of the Chl fluorescence rise was reduced by ~36% in *NFR-3* and by ~53% in *NFR-13,* with respect to WT (Table 1).

These results were supported by quantification of LHC protein content. The abundance of selected photosynthetic subunits was assessed by immunotitration on cell extracts and expressed relative to WT, upon normalization to the PSII inner antenna CP43 content (Figure 1B). The LHCII/PSII ratio was reduced in both *NFR* mutants (−68% vs. WT), while the PSI/PSII (PsaA/PsbC) ratio was significantly higher in mutants. When cultivated under limiting light conditions (70 µmol photons m^−2^ s^−1^), both mutants showed a significant decrease in growth rate and a lower mean biomass production with respect to WT, consistent with the reduction in PSII antenna size (Figure S3).

The content in LHCII, PsbC and PsaA subunits was quantified on a per cell basis by immunotitration, and expressed as a percentage of the corresponding WT values. Figure 1C shows that LHCII content in *NFR* cells was significantly reduced vs. WT, while PSI and PSII contents were less affected.

The operation efficiency of the photosynthetic machinery was investigated in a wide range of irradiances, by measuring the light-saturation curve of photosynthesis in WT and *NFR* cells, grown under photoautotrophic conditions (Figure 1C). The rate of O_2_ production rose as a function of light intensity within the range 0–1000 µmol photons m^−2^ s^−1^, showing a linear increase for WT and *NFR* lines at irradiances below 150 µmol photons m^−2^ s^−1^.

The linear regressions of O_2_ release vs. light intensity exhibited a slope of 0.71 ± 0.07 for WT, with a significant increase in *NFR* strains (1.27 ± 0.13 in *NFR-3*, 1.94 ± 0.29 in *NFR-13*), suggesting that the effective quantum yield of photosynthesis was enhanced in *NFR* mutants with respect to WT. Half-saturation of photosynthesis was reached at similar light intensity in WT and *NFR-3* strains (approximately 110–130 µmol photons m^−2^ s^−1^), while it was significantly higher in *NFR-13* strain (Table 3). Values of P_max_, the maximum rate of light-induced O_2_ evolution (photosynthesis net respiration), was reached around 2000 µmol photons m^−2^ s^−1^ in WT strain, with a value of 96 ± 5 µmol O_2_ mg Chl^−1^ h^−1^; this value was significantly higher in *NFR* cells (225 ± 2 in *NFR-3*, 323 ± 45 in *NFR-13*). O_2_ production rate was normalized on Chl content, yielding P_max_ as a measure of the productivity for the two strains per unit Chl. The dark respiration rate on a per cell basis was the same in WT and mutant strains (Table 3). As a whole, these results point to an enhanced photosynthetic productivity of *NFR* mutants with respect to the WT.

### 2.3. Phototolerance of WT and NFR Strains during EL Exposure

EL conditions lead microalgae to photooxidation: the enhanced release of ^1^O_2_ [44] results in lipid peroxidation, Chl bleaching and a consequent decline in the photosynthetic yield. Photoautotrophs have evolved a plethora of mechanisms aimed at limiting photodamage; these include the tight regulation of Car pool size, which is increased in response to the EL stress [45]. Therefore, the mutant strains *NFR*, due to a constitutive enhancement of Car content, are expected to counteract photooxidative conditions more efficiently, thus limiting photoinhibition.

**Figure 2:**
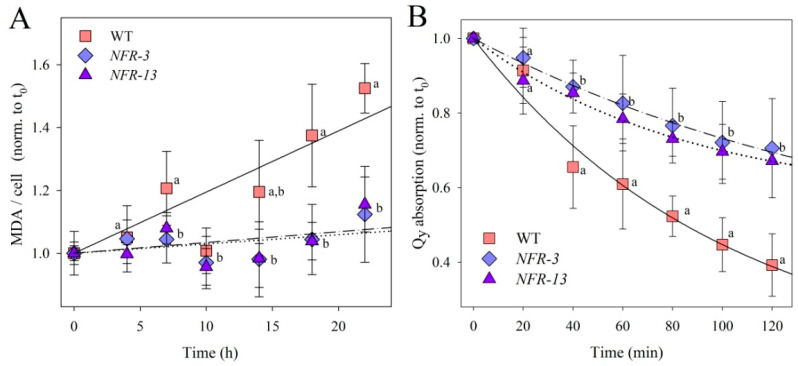
Photooxidation of *C. vulgaris* WT and *NRF* mutant genotypes under photooxidative stress. (**A**) Cell suspensions of WT and mutant strains were treated with 1400 µmol photons m^−2^ s^−1^ at 20 °C, and kinetics of malondialdehyde (MDA) formation were followed. MDA is an index of membrane lipid peroxidation, and was quantified by HPLC as thiobarbituric reactive substances. Slopes of the linear fit (proportional to the rate of MDA release) were 0.0195 ± 0.0056 (WT), 0.0029 ± 0.0024 (*NFR-3*), 0.0034 ± 0.0031 (*NFR-13*). (**B**) Cell suspensions were treated with strong white light (14,000 µmol photons m^−2^ s^−1^, 20 °C) and the amount of Chl was evaluated by measuring the absorption area in the region 600–750 nm. See Materials and Methods for details. Symbols and error bars show means ± SD, *n* = 4. Values marked with the same letters are not significantly different from each other within the same time point (ANOVA followed by Tukey’s post-hoc test at a significance level of *p* < 0.05).

To characterize the photooxidative stress experienced by WT and *NFR* mutants, cells were transferred from control conditions to strong irradiance (Figure 2). A well-known consequence of ROS release in the thylakoids is the peroxidation of unsaturated lipids, a reaction that releases malondialdehyde (MDA) as a by-product. When cultures were exposed to 1400 μmol photons m^−2^ s^−1^, at 24 °C for 24 h, rate of MDA release was ~5 times higher in WT cells with respect to *NFR* strains (Figure 2A), implying a significantly lower level of oxidative damage of the membranes in the mutants.

We then analyzed cell suspensions of WT and mutant strains, by a time-course pigment bleaching upon transfer of cultures from low to extremely strong light (14,000 μmol photons m^−2^ s^−1^, at 20 °C). In all cultures, the total cell Chl content decreased progressively upon EL treatment (Figure 2B); however, the rate of Chl bleaching was twice faster in WT respect to *NFR* strains.

To further investigate the photooxidative stress experienced by these genotypes, liquid cultures were grown photoautotrophically under very high irradiance (2500 μmol photons m^−2^ s^−1^, 24 °C), for 6 days in batch conditions. While the WT strain showed a long lag phase, *NFR* mutants exhibited a robust early growth phenotype, with *NFR-13* culture displaying the faster growth rate (Figure S4). Among the mechanisms possibly underlying the higher biomass yield of *NFR* is the reduced optical density of culture, which allows for more homogeneous light distribution within the culture: indeed, pale-green mutants of different algal species [46,47,48] possess an enhanced photosynthetic productivity with respect to the corresponding WT strain. Therefore, the *C. vulgaris* mutant *PG-14* previously described [36] was included in the growth trial as internal control. The *PG-14* mutant showed a growth kinetic and final biomass yield higher than WT (Figure S4), consistent with previous report [36]. Growth enhancement of *NFR* strains with respect to WT was higher than with *PG-14* under photooxidative conditions, thus suggesting higher productivity of *NFR* strains was caused not only by enhanced light diffusion in the culture, but also by higher tolerance to EL stress.

### 2.4. Biomass Productivity of WT and NFR Strains in Laboratory-Scale PBR

**Figure 3:**
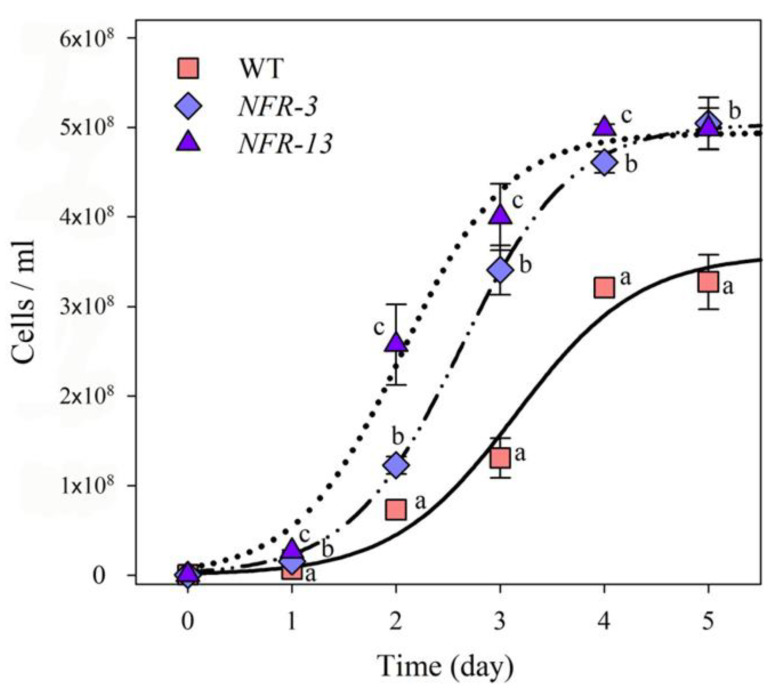
Growth curves of WT and mutant strains. Growth of WT and *NRF* mutant lines was performed under photoautotrophic conditions, in 1-L cylinders, illuminated with 1400 µmol photons m^−2^ s^−1^, 25 °C. Cultures were maintained in a semi-batch system fed with air/CO_2_ mix; CO_2_ supply was modulated in order to keep the pH of the medium always between 6.8 and 7.2. Symbols and error bars show means ± SD, *n* = 5. Values marked with the same letters are not significantly different from each other within the same time point (ANOVA followed by Tukey’s post-hoc test at a significance level of *p* < 0.05).

The above results are consistent with a higher tolerance to photooxidative damage of *NFR* strains with respect to WT, which implies mutant strains could have an improved biomass productivity in PBR dense cultures under elevated irradiances. Therefore, strains were cultivated photoautotrophically over a period of 6 days in a laboratory-scale PBR, a semi-batch cultivation system consisting of 1 L glass cylinders. Cells were grown in minimal medium (BG-11), illuminated with 1400 μmol photons m^−2^ s^−1^ at 24 °C, under a day/night cycle of 16:8 h. Each cylinder was fed with a mix of air and CO_2_, whose relative abundance was tuned in order to maintain the pH of the medium between 6.8 and 7.2. Both *NFR* mutants showed a faster growth rate with respect to WT (Figure 3), and cell concentration at day 6 was about 5 × 10^8^ cells mL^−1^ in both mutants vs. 3.6 × 10^8^ cells mL^−1^ in the WT (Figure 3).

The specific growth rate (μ) for *NFRs* was higher than that obtained for the WT strain (Table 4). Furthermore, *NFR* mutant strains reached a biomass productivity of 0.56 g L^−1^ day^−1^, which was significantly improved (+23%) with respect to the yield obtained in WT (0.43 g L^−1^ day^−1^, see Table 4).

**Figure 4:**
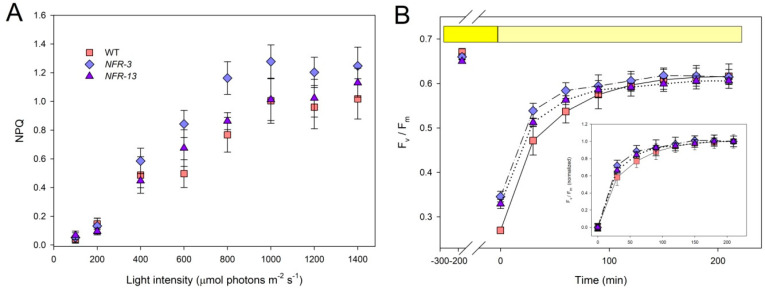
Analysis of Chl fluorescence during photosynthesis under EL. (**A**) Chl fluorescence was monitored in cultures dark-adapted for 2 h. Cell suspensions were illuminated for 25 min and the non-photochemical quenching (NPQ) was determined at the end of light treatment (i.e., during steady-state photosynthesis). (**B**) PSII quantum yield recovery after EL treatment was quantified on WT and *NFR* strains by measuring F_v_/F_m_ recovery in low light (20 μmol photons m^−2^ s^−1^, 24 °C, light yellow bar) upon 3 h of EL treatment (1800 μmol photons m^−2^ s^−1^, 24 °C, yellow bar). (*Inset*) kinetics of F_v_/F_m_ were zeroed at the end of the EL treatment and normalized to the maximum F_v_/F_m_ during low light recovery. Data are expressed as mean ± SD, *n* = 4.

The higher photoresistance of *NFRs* could be ascribed to a number of mechanisms, which include the photoprotective Non-Photochemical Quenching (NPQ). NPQ catalyzes the safe dissipation of the excitation energy in excess as heat, and its amplitude is modulate by the size of the xanthophyll pool [49,50]. Once assessed at steady-state photosynthesis, NPQ amplitude showed negligible differences between WT and *NFR* strains, over all the light intensities tested (Figure 4A and Figure S4). Alternatively, resistance and/or recovery from photoinhibitory EL treatment could be improved in mutant strains. To verify this possibility, we treated cells for 3 h at 1800 µmol photons m^−2^ s^−1^, 24 °C, and measured F_v_/F_m_ ratio. The extent of PSII photoinhibition was significantly higher in WT vs. *NFR* strains, with F_v_/F_m_ values being 15% lower at the end of light treatment (Figure 4B); this is consistent with the milder oxidative damage experienced by the mutants (See Figure 2). However, recovery of F_v_/F_m_ in low light (Figure 4B, inset) showed that all genotypes performed equally, implying that the higher productivity of *NFR* strains was not due to a strengthened PSII repair system.

### 2.5. Lipid Productivity under Nitrogen Starvation

Microalgae gained increasing interest as a feedstock for biofuel production. Thus, an analysis of oil content in WT and mutant strains is crucial to assess their potential for commercial-scale oil production. EL [51] and nutrient deficiency [52] were recognized as stress factors which promote lipid accumulation in microalgae. Signaling involves photooxidative stress perception, while ROS and ROS-related by-products likely represent effector molecules, as suggested by the correlation between ROS level and lipid content in *C. vulgaris* cells [53]. It comes that the higher resistance to photooxidative damages, measured in *NFR* strains, might impair oil yield by affecting ROS-based crosstalk signals. Thus, the total lipid content was determined in WT and *NFR* strains. Microalgae were first grown in lab-scale PBR (see Figure 3) in nutrient-rich BG-11 medium, to attain high cell density, then were transferred to the same medium except that N was limiting (−95% N content than standard BG-11). After 11 days of growth, lipid content per DW was determined gravimetrically (Table S1). Lipid content in WT algae was about 25% of the dry biomass, while cells from both *NFR* lines had a significantly lower lipid fraction, in the amount of 16% of the dry biomass.

### 2.6. Genotypic Characterization of NFR Strains by Whole-Genome Sequencing

The complex regulation of EL adaptation mechanisms is poorly understood in microalgae and most key factors are still awaiting elucidation. Mutant strains represent valuable tools to identify the underlying genotypic traits. Therefore, the two high Car content mutants, as well as the corresponding WT strain, were investigated in a whole-genome re-sequencing approach in order to identify the genetic background of the phototolerant phenotypes. For the systematic identification of the mutation(s) responsible for the Car-accumulating phenotypes, we applied a workflow based on open source bioinformatics tools. Sequencing the parental WT strain alongside *NFR* mutants enabled us to identify and remove common variants present in the WT background strain. Whole-genome sequencing was performed on an Illumina HiSeq platform. After trimming and quality filtering, the mean obtained coverage was 65.9X, 55.2X and 18.6X for the WT, *NFR-3* and *NFR-13* respectively. Percentage of the reads mapped to the reference genome were 99.4, 71.5, and 60.8% for WT, *NFR-3*, and *NFR-13,* respectively (Table 5).

After the filtering process and manual inspection with Integrated Genomics Viewer (IGV), we detected a total of 34 variations (SNPs and small INDELs, see also Figure S5) that differed between the mutants and parental WT. Variants are listed in Table 6, and discussed further ahead.

## 3. Discussion

Realization of microalgae potential to satisfy global demands requires a decrease of the current production costs for biomass. Among factors contributing to the price of bio-products purified from microalgae are energy and material inputs, as well as cellular content of the desired molecule [54]. It comes that cost-effective production needs selection of strains with enhanced accumulation of a valuable compound. Moreover, productivity of the most common industrial strains is far lower than maximal theoretical estimations, suggesting that removing bottlenecks which limit biomass yield is crucial in strategies aiming to make algal biofactories profitable on an industrial scale.

### 3.1. Optimization of Light-Use Efficiency Enhances Biomass Yield

The inefficient use of light, due to high optical density of cultures, is among the factors which limit algal biomass production in the artificial conditions of a PBR. Indeed, it was shown that the biomass yield in PBRs increased with decreasing the light path, which allows for more homogeneous light distribution in the culture vessel [55]. Unfortunately, short light paths in flat-panel PBR limit the overall volume of culture and, thus, the economic viability of large-scale plants.

A number of studies have shown that biomass yield can be improved by optimizing optical features of algal cultures through genetic engineering approaches: targeting genes such as *TLA1*, which control antenna size in *Chlamydomonas reinhardtii*, resulted in higher biomass yield [56]; random mutagenesis applied to species with a high industrial interest such as *C. sorokiniana* and *N. gaditana*, selected pale-green mutants which exhibited increased light-use efficiency [47,48]. A similar approach was used in this work with *Chlorella vulgaris*, searching for low optical density traits to increase light penetration and light-to-biomass yield, in order to find out a background strain for further domestication projects.

Both *NFR* mutants are defective in PSII antenna size, consisting of a LHCII content of 31% (in *NFR-3*) and 32% (in *NFR-13*) with respect to WT (Figure 1B). Fluorescence induction in cells treated with DCMU confirmed that both *NFR* strains had a reduced PSII functional antenna size (Figure 1A).

Photosynthetic rate was significantly enhanced in both *NFR* strains; yet this effect was larger in the strain with lower cross-section (Table 3): indeed, the P_max_ of *NFR-3* was 134% larger than in WT at saturating irradiances, while that of *NFR-13* increased by 236%, implying the reduction in antenna size enhanced photosynthetic yield. These results are consistent with those reported for *C. reinhardtii tla1* and *tla3* strains with truncated antenna system [46,56].

Photooxidative stress caused by EL conditions is a major reason for yield reduction in algal cultures [57]. Microalgae exposed to saturating light intensity undergo PSII damage, which must be repaired by de novo synthesis [58]. The process impairs the overall efficiency of light-to-biomass conversion efficiency, therefore, it should be minimized to achieve high productivity.

Moreover, cells in dense suspensions are rapidly mixed and experience a rapid shift from darkness to saturating light, and mixing rate on a ms time-scale can easily be achieved in tubular PBR [59]. The consequent fluctuations in light intensity are faster than mechanisms required for counteracting EL stress, such as the activation of dissipative channels or the reversible phosphorylation of LHCII. Therefore, the repeating fluctuations between low and high light phases challenge the light acclimation capacity of the photosynthetic apparatus [60]. In this work, growth rates of cultures were measured in the long term under strong irradiance (2500 μmol photons m^−2^ s^−1^) in batch conditions (Figure S4). During 6 days of growth, the *PG-14* strain we used as a reference with reduced antenna size and without Car content enhancement [36] showed a 57% increase in productivity with respect to WT (see Figure S3B), meaning that a reduction of LHC per PSII by 40% (previously determined for this genotype) significantly improved the phototolerance of *C. vulgaris* cells. Interestingly, *NFR* mutations showed a further increased growth rate with respect to both WT and *PG-14* under EL. We conclude that overaccumulation of Car and diminished optical density of the cultures have a synergistic effect in improving light-use efficiency under strong irradiance.

As a consequence of the highly reactive excited states generated during oxygenic photosynthesis, algae have evolved a number of photoprotective mechanisms to limit damage to cell structures [61]. These mechanisms are effective in (i) regulating either ^1^Chl* [22] or ^3^Chl* state formation [35], thus, ROS release, or (ii) scavenging ROS [62]. The first group includes the energy dissipation events which diminish the excitation pressure on PSII, while the antioxidant network comprises scavenging enzymes and antioxidant molecules [63], including the thylakoid-bound antioxidants Car which catalyze both quenching of ^1^Chl* and ^3^Chl* and scavenging of ^1^O_2_.

NPQ is the main mechanism that regulates ^1^Chl* lifetime [64,65], making it a candidate process underlying higher biomass yield of *NFR* strains. However, NPQ amplitude, once measured over a range of irradiances, was essentially the same in WT and *NFR* genotypes (Figure 4A), implying that differences in growth cannot be ascribed to changes in Chl quenching activity. A similar consideration applies to the activity of PSII repair (Figure 4B).

The phototolerance of *NFR* mutants was significantly higher vs. WT. The observed lower lipid peroxidation and higher resistance to Chl photobleaching (Figure 2) suggest either a reduced ROS release or a more efficient scavenging activity of ROS in the chloroplasts. The latter appears as the most likely hypothesis, as the content in the Car antioxidants is far higher in *NFR* strains (Table 1). Indeed, mutants impaired in the accumulation of Cars, in both plants and algae, were unable to acclimate to high irradiance, and progressively lost photosynthetic capacity along with oxidation of pigment–protein complexes and accumulation of ROS [66,67].

In addition to enhanced antioxidant capacity, other mechanisms could improve light-to-biomass conversion efficiency in *NFR* strains. We expect a lower ROS release in mutant lines due to higher antioxidant capacity. Depending on its concentration, ^1^O_2_ was shown to play a role as second messenger in cell signal transduction pathways, controlling a number of stress-response mechanisms [68]; e.g., in *C. reinhardtii*, specific promoter regions mediate transcriptional responses to ^1^O_2_ [69,70], while experience of a moderated ^1^O_2_ level was shown to improve resistance of algal cells to harsher photooxidative stress [68].

A number of plastid-derived signals were shown to be involved in the adaptation of cells to the light environment, including cleavage products of Car or intermediates of Car biosynthesis [71]. Indeed, under EL stress, oxidized by-products of β-carotene, such as β-cyclocitral (β-CC) and β-ionone, work as signaling molecules in plants, enhancing resistance to EL and other stresses [72,73]. Release of Car cleavage products was also described in cyanobacteria [74], thus suggesting these signal pathways are ubiquitous in the green lineage.

Interestingly, previous work showed that resistance to oxidative stress can also be obtained without any increase in Car/Chl ratio; this is the case of ^1^O_2_-resistant (SOR) strains [36], the photoprotection capacity of which was significantly enhanced than WT strain, despite the pool of Car not being differentially affected in these genotypes upon light stress. However, SOR strains, when cultivated in the same lab-scale PBR here reported, at 1400 µmol photons m^−2^ s^−1^, stood out for higher mean biomass productivity than both *NFR* strains. These *C. vulgaris* genotypes can be ranked with respect to the light-to-biomass conversion efficiency as follows: WT < *NFRs < SOR-5 = SOR-6* [36].

In the condition of prolonged EL treatment of cultures tested here, our results point to a specific involvement of Car in the defense of the photosynthetic machinery against photooxidative damage. Data also imply that multiple mechanisms take part in photoprotecting algal cells: indeed, the EL resistance measured in SOR strains was not related to any increase in the size of the Car pool.

### 3.2. Influence of ROS-Resistance Traits on Stress-Induced Lipid Production

While sustained ROS release under EL causes lipid peroxidation, mild photooxidative stress conditions enhance lipid accumulation in oleaginous microalgae [75]. The mechanism possibly underlying such response is a ROS signaling network that up-regulates the pathway of fatty acid biosynthesis [76]. Consistently, in *C. vulgaris*, a positive correlation between oil content and ROS level was found [53], while in *S. cerevisiae* the endoplasmic reticulum triggers development of lipid bodies upon perception of oxidative stress [77]. Overall, evidence for a correlation between intracellular ROS level and abundance of TAGs is still limited and molecular details are missing. In the case of *NFR* strains, if ROS level promotes fatty acid biosynthesis by complex signaling pathways, then we might expect an inhibiting effect in the accumulation of oil due to the enhanced antioxidant activity of the mutants. Indeed, quantification of intracellular lipid content, upon short-term nitrogen-starvation treatment of cultures, supports this hypothesis: oil content was significantly reduced in both *NFR* strains (−35% vs. WT, see Table S1). Alternatively, such a change in lipid content could be a consequence of the missense amino acid substitution in Glycerol-3-phosphate acyltransferase 3 (Table 6, Figure S7), which is indeed involved in the terminal steps of triacylglycerol biosynthesis in the endoplasmic reticulum (see below).

### 3.3. NFR as Promising Car Producer Strains for Commercial Applications

The demand for nutraceuticals has been increasing over the last years. Microalgae contribute as being rich in proteins and bioactive compounds such as essential fatty acids, Car and vitamins. However, only a few microalgal species are currently commercialized as nutraceuticals [7]. *Chlorella vulgaris* is among the few species approved by the European Food Safety Authority for human consumption. *Chlorella* biomass is widely used as food supplement or to enhance the nutritional value of food products [78]. However, organoleptic features of algal biomass, e.g., strong green color or grassy taste, might disappoint the consumer, preventing them from including supplemented food in their diet [79]. Isolation of mutant strains with altered pigmentation, e.g., higher Car/Chl ratio, does represent an alternative strategy for improving the qualities of algal biomass as food additive. Furthermore, forward genetic approach is of particular relevance in the food industry, since it avoids restrictions to GMO [80].

In this work we developed two Chl-deficient strains of *Chlorella vulgaris* of interest for nutritional applications. The changes in color of *NFR-3* and *NFR-13* mutant strains in N-limited conditions (Figure S1) are due to a lower Chl content, and to a 70% and 146% increase in Car content, respectively, with respect to the standard BG-11 medium. Under photoautotrophic growth, both strains showed a significantly improved growth performance with respect to WT, reaching a concentration of 3.79 g FW L^−1^ (*NFR-3*) and 4.74 g FW L^−1^ (*NFR-13*) in batch conditions. Moreover, the improved growth rates were maintained even when scaled up in 1-L lab-scale PBR (Figure 3, Table 4), a result which support their feasibility as potential candidates for the food industry and for the development of novel food supplements.

The strains here described point to *C. vulgaris* as an attractive candidate for the production of xanthophylls: *NFR-13* strain showed a volumetric lutein content of 40 mg L^−1^ under N-starvation, significantly higher than those reported for other lutein-producing strains grown under photoautotrophic conditions [45,81]. Moreover, *NFR-13* cultures showed a high specific growth rate (0.08 h^−1^), thus, it represents a promising strain for the production of xanthophylls for commercial applications.

### 3.4. Whole-Genome Sequencing to Identify Gene(s) Responsible for the Enhanced Light-Use Efficiency in C. vulgaris

The phenotype of *NFR* strains is the product of one or more mutations which affect regulatory and metabolic processes of Car biosynthesis. Here, we identified genetic variants among which these mutations can be searched for, and suggest how these variations could alter gene function, yielding the observed phenotype of *NFR-3* and *NFR-13* mutants.

1. Glycerol-3-phosphate acyltransferase 3. A transversion (G to C) was observed at nucleotide 534 on the 6th exon. This results in missense amino acid substitution of a glutamic acid into an aspartic acid, a mutation shared by both *NFR* mutants, affecting a subunit involved in neutral lipid metabolism. Glutamic acid and aspartic acid are both acidic amino acids with side chains containing a carboxylic acid group which carries a full negative charge. In terms of interaction modes, both amino acids include ionic, van der Waals and H-bonds. However, glutamic acid differs from aspartic acid only in that its side chain is longer by one methylene group. This results in higher side chain flexibility in glutamic acid compared to aspartic acid whose side chain flexibility is moderate. Transmembrane topology prediction indicates that the site of mutation is at the boundary between cytoplasmic domain (residues 169–179) and the consecutive transmembrane region (residues 180–199). Multiple sequence alignments of top BLAST hits indicate that the last two residues of the cytoplasmic domain (E178 and R179) are conserved among green cut species (Figure S7). Glycerol-3-phosphate acyltransferase 3 is a key component of the diglyceride-3-phosphate synthesis pathway. Consistently, its activity has been reported to be directly correlated to the amount of glycerophospholipids in *E. coli* cells [82] and used as a target for membrane engineering to enhance production and accumulation of ß-carotene [83].
2. E3 Ubiquitin-protein Ligase SP1-related (PTHR47568:SF2) encoding sequence contains a splice donor variant and is shared by both mutants, *NFR-3* and *NFR-13*. A transition (G to A) occurs at the first nucleotide of the second intron affecting canonical GT-AG splice site pairs. Group of E3 ubiquitin–protein ligases, including SP1 from *Arabidopsis* (UniProtKB: Q8L7N4), are involved in regulation of plastid’s proteome via ubiquitination and subsequent degradation of translocon at the outer envelope membrane of chloroplasts (TOC) complexes [84]. In addition, it promotes stress tolerance by depleting the chloroplast protein import apparatus, which limits photosystem assembly and the potential for ROS formation [85]. Formighieri et al. [86] demonstrated that absence of ARSA1, a protein localized in the *C. reinhardtii* cytosol, led to a strongly decreased Chl content per cell; ARSA1 was shown to be required for optimal biogenesis of photosystems due to its involvement in the accumulation of TOC34, a component of the outer chloroplast membrane translocon complex. Interestingly, in both species, a mutation targeting a component interacting with TOC is associated with a pale green phenotype.
3. Serine/threonine-protein phosphatase 2A 65 kDa regulatory subunit A contains variation observed in *NFR-13* mutant only. Transition mutation (C to T) results in substitution of an arginine by cysteine. 2A protein phosphatases are evolutionarily conserved and carry out multiple functions such as growth- and stress-related signaling, cell cycle regulation, vesicle trafficking, as well as regulation of the activities of a number of enzymes involved in key metabolic pathways [87]. The mutant 2A protein phosphatase in *NFR-13* contains fatty acid desaturase domain (IPR005804) which is involved in the lipid metabolic process (GO:0006629).
4. Both *NFR-3* and *NFR-13* share an intronic variation on Allophanate hydrolase (g7748). In *Chlamydomonas reinhardtii*, allophanate hydrolase is involved in urea hydrolysis to produce ammonium depending on the source of nitrogen available [88].
5. Another exonic mutation found only in *NFR-3* targets biosynthetic arginine decarboxylase (g9015) involved in the polyamine biosynthesis pathway. In plants, polyamine accumulation was shown to correlate with Car content [89].
6. A missense amino acid substitution (Arg233Cys) was identified on S49 family peptidase (g6424) in *NFR-3*. The predicted protein contains peptidase_S49 domain (IPR002142) from residues 20–236. InterPro entry (IPR002142) describes proteolytic enzymes that exploit serine in their catalytic activity. The sequences of these ubiquitous enzymes are variously annotated in different taxonomic groups. In plants, these proteolytic sequences are annotated as Signal peptide peptidase A (SppA; protease IV; MEROPS identifier S49.001) which are involved in cleavage of signal peptides.
7. *NFR-13* mutant contains a point mutation resulting in Ala80Thr on Acylamino-acid-releasing enzyme-like (g7937). Acylamino-acid-releasing enzymes have been identified and characterized in plants. They are found to be mainly localized in the stroma of chloroplasts, and are possibly involved in degradation of glycated/oxidized proteins, such as glycated RuBisCO (ribulose-1,5-bisphosphate carboxylase/oxygenase), and thus contribute to sustain the chloroplast antioxidative system [90].

Lastly, 6 out of 34 identified variants lie within or in close proximity to gene bodies containing transcriptional or translational regulatory domains, namely, transcription initiation factor IIB-2 (g501, *NFR-3*), Nuclear/nucleolar GTPase 2 (g672, *NFR-13*), Nuclear transport receptor (g671, NFR-13), S1 motif domain-containing protein (g8549, *NFR-3/NFR-13*), U3 small nucleolar ribonucleoprotein protein IMP4 (g8749, *NFR-3*). In *NFR-13*, an amino acid substitution (Arg150Cys) on an RNA helicase (g5804) with U5 small nuclear ribonucleoprotein 200 kDa helicase domain (PTHR24075:SF5) is identified. This predicted protein is likely to be involved in spliceosome assembly, activation and disassembly (inferred from sequence similarity to UniProtKB:P32639).

## 4. Conclusions

Here we show that *C. vulgaris* mutagenesis and selection in the Car biosynthesis inhibitor norflurazon resulted in a significant increase in the size of the Car cellular pool. The mutant strains were more tolerant to EL conditions, as shown by reduced lipid peroxidation and pigment bleaching. Stress protection is probably due to the function of Car in preventing oxidative damage of membranes. Moreover, these strains showed an improved biomass productivity with respect to WT in mass cultures under EL conditions. Overall, our results show that a domestication strategy, focused on the modulation of both cell optical density and resistance to photooxidative stress, succeeded in the development of improved strains, which is of interest for industrial production. Although our genomic analysis cannot unequivocally identify the genetic origin of the phenotypes reported for *NFR* genotypes, we can tentatively propose that the mutation on Glycerol-3-phosphate acyltransferase 3, which is shared by *NFR-13* and *NFR-3* mutants, might be involved in the reduced accumulation of lipids upon N starvation (Table S1). Similarly, the mutation at the E3 Ubiquitin-protein Ligase SP1 might cause the pale green phenotype through its effect on TOC complex assembly. Experimental verification of these hypotheses will involve targeting these individual genes by either genome editing [91] or tapping from the mutant collection [92] in the green algae relative species *Chlamydomonas reinhardtii*.

## 5. Materials and Methods

*Strains and culture conditions. Chlorella vulgaris* WT strain was obtained from the SAG Culture Collection of Algae (Goettingen University, Germany, http://www.uni-goettingen.de/en/catalogue-of-strains/185049.html (accessed on 1 May 2021)) as SAG strain number 211-11p. Cells were maintained on TAP agar plates [93] and grown in either minimal (BG-11) [94] or rich (TAP) liquid media. Shaken flasks (120 rpm) and plates were illuminated from the top with 70 μmol photons m^−2^ s^−1^, photoperiod of 16/8 h light/dark, 25 °C (control condition); irradiance was provided by warm-white LEDs (Epistar 35mil Chip High Power LED, warm white LEDE-P20B-DW, Wayjun Tech., Shenzhen, China). For nitrogen starvation experiments, cells were grown in the BG-11 medium containing excess nitrogen source (NaNO_3_ 17 mM); they were then collected by centrifugation at the end of the logarithmic growth phase, washed twice with sterile water, and re-suspended in a modified BG-11 medium with limiting N source (NaNO_3_ 0.8 mM). For physiological measurements, cultures were harvested during the logarithmic growth phase (about 1–3 × 10^7^ cells mL^−1^).

*Mutagenesis and screening protocols.* Liquid cultures of *C. vulgaris* WT strain were harvested by centrifugation at the exponential phase of growth (~1 × 10^6^ cells mL^−1^), re-suspended in fresh BG-11 medium to 5 × 10^7^ cells mL^−1^, and treated with 2.2% (*w*/*v*) of *ethyl methanesulfonate* (EMS). The survival curve for mutagenesis with EMS was carried out to determine the mutagen concentration which resulted in around 5–10% of cell viability. Upon 2 h maintenance in the dark, to prevent light-activated DNA repair, cells were plated at 100-fold dilution on minimal medium, and the inhibitor norflurazon (NF) was used as selection method. NF is a herbicide inhibiting the carotenogenic enzymes phytoene desaturase [39]. A wide range of concentrations of the chemicals were previously tested to find out the minimal concentration which inhibited grown of the WT strain. For the selection of NFR (norflurazon resistant) strains, mutagenized cells were spread on a solid minimal medium containing 4 μM NF and incubated at 70 μmol photons m^−2^ s^−1^ for 2 weeks; single colonies appeared after 14 days.

Cells were subjected to two rounds of random chemical mutagenesis with EMS. In a first selection step, lines were selected for pale green phenotype by direct sight inspection, then pale green strains were pooled, grown to enhance cell number, submitted to a second treatment with EMS, and mutants were selected for resistance to inhibition of Car biosynthesis.

Selected lines upon two rounds of mutagenesis were inoculated onto fresh minimal medium, grown in the light for seven days, and the Chl content per cell was determined. The herbicide-resistant colonies were sub-cultivated several times in liquid BG-11 medium containing 4 μM NF to check their resistance to the herbicide and further analyzed for Car and Chl content per cell and for growth rate.

*Cell count and pigment analysis*. Cell density was measured using an improved Neubauer hemocytometer. Pigments were extracted from intact cells with 100% dimethyl-formamide (DMFA). The supernatant of each sample was recovered after centrifugation (10 min at 15,000× *g*, 4 °C), and pigments were separated and quantified by HPLC [95].

*Gel Electrophoresis and Immunoblotting*. For SDS-PAGE and immunotitration analysis, cells were resuspended in Loading Buffer (5% glycerol, 1% SDS, 2.5% 2-mercaptoethanol, 0.1 M Tris, 0.1 M Tricine pH 8.45) and ground in a tissue homogenizer (Precellys, Bertin, France) by adding a ceramic lysing matrix. The supernatant of each sample was recovered after centrifugation (10 min at 15,000× *g*, 4 °C) and Chl content of extracts was determined by fitting of the spectra of acetone extracts with the spectra of individual pigments [96]. SDS-PAGE analysis was performed with the Tris-Tricine buffer system [97]. For immunotitration [98], a range of total protein extract corresponding to 0.25–1.0 µg of Chl were loaded for each sample and electroblotted on nitrocellulose membranes (Amersham Protran^®^, pore size 0.45 µm). Proteins were detected with primary antibodies (home-made, α-CP43 and α-LHCII; from Agrisera, α-PsaA AS06-172-100) and an alkaline phosphatase-conjugated secondary antibody (Sigma-Aldrich A3687). Signal amplitude was quantified using the GelPro 3.2 software (Bio-Rad).

*Measurements of photosynthetic activity*. The oxygen evolution activity of the cultures was measured at 25 °C with a Clark-type O_2_ electrode (Hansatech, UK) upon illumination with white light provided by a halogen lamp (Schott, Germany). Samples of 2 mL cell suspension (5 × 10^6^ cell mL^−1^) were loaded into the oxygen electrode chamber; 3 mM NaHCO_3_ was added to the cell suspension prior to the O_2_ evolution measurements to ensure electron transport was not limited by the carbon supply.

*In vivo chlorophyll fluorescence analysis.* Fluorescence induction kinetics were recorded with a home-built apparatus, as previously described [99]. Variable fluorescence was induced with a green light of 15 μmol photons m^−2^ s^−1^ at RT, on cells suspensions (1 × 10^7^ cells mL^−1^) in BG-11 medium containing 50 µM DCMU. The reciprocal of time corresponding to two-thirds of the fluorescence rise (T_2/3_) was taken as a measure of the PSII functional antenna size [42,43]. Maximum quantum efficiency of PSII (F_v_/F_m_) [100] was measured on cell suspension with a PAM 101 fluorimeter (Heinz-Walz, Effeltrich, Germany). The light dependence of NPQ was measured through Chl fluorescence with a Fluor-Cam 700MF (PSI, Drasov, Czech Republic), on cell suspension dark-adapted for 2 h at RT; NPQ was assessed at steady-state photosynthesis, upon 25 min illumination over a range of light intensities (100–1400 µmol photons m^−2^ s^−1^).

*Determination of the sensitivity to photooxidative stress.* The extent of lipid peroxidation in cells was estimated by measuring *malondialdehyde* (MDA) formation, as an indirect quantification of lipid peroxides. Quantitative evaluation was done by transferring aliquots of WT and mutant cell suspensions in BG-11, corresponding to 4 × 10^6^ cells mL^−1^, into a 24-well culture plate, 2 mL total volume in each well, kept on a rotary shaker and illuminated for 2 days with high light (1400 µmol photons m^−2^ s^−1^, 20 °C). Samples (2 × 10^5^ cells) were taken for analysis for a period of 48 h, and frozen in liquid nitrogen. MDA content of aliquots was quantified as previously described [101].

The photobleaching kinetics of Chl cell content were measured on cell suspensions (2 × 10^6^ cells mL^−1^, in BG-11 + 0.03% *w/v* agarose) using actinic light intensities of 14,000 μmol of photons m^−2^ s^−1^ for 2 h; temperature of samples was maintained at 20 °C. During the illumination, the absorbance area between 600–750 nm was recorded; the initial and maximal absorbance were set, so the same absorbance area was used in the wavelength range 600 nm < λ < 750 nm for all the samples.

*Growth analysis.* Growth experiments were performed at 25 °C in a home-built photobioreactor, composed of glass cylinders with a maximum light path of 8 cm and a working volume of 1 liter each [47]. Cultures were continuously mixed with a flux of air and CO_2_. The ratio of compressed air and CO_2_ was automatically adjusted to keep the pH of the medium within the range of 6.8–7.2. Each autotrophic batch cultivation was carried out in duplicate. Illumination was provided by a panel of warm-white LEDs (Epistar 35mil Chip High Power LED, warm white LEDE-P20B-DW), microalgae were exposed to irradiance of 1400 µmol photons m^−2^ s^−1^, with a photoperiod of 16/8 h light/dark; batch cultivation was carried out in duplicate.

For cultivation under very strong irradiance (2500 µmol photons m^−2^ s^−1^), illumination was provided by a panel of warm-white LEDs (Epistar 35mil Chip High Power LED, warm white LEDE-P20B-DW), microalgae shaked in flasks were exposed to an irradiance of 2500 µmol photons m^−2^ s^−1^, with a photoperiod of 16/8 h light/dark; batch cultivation was carried out in triplicate. The parameters determined to monitor cell growth were cell number and dry biomass weight, for which the washed cell pellets were dried overnight in a lyophilizer.

*Determination of total lipid content.* Total lipids were extracted from 100 mg homogenized biomass (4 cycles of 30 s at 8000 rpm, with a Precellys homogenizer, Bertin, France) using an extraction protocol by [102], with a total of 3 mL methanol, 6 mL chloroform, and a subsequent washing step with 4 mL water. Net total lipid amount was determined gravimetrically.

*Whole-genome sequencing and identification of unique mutations.* For DNA preparation, 2 × 10^8^ cells were collected by centrifugation, then resuspended in 1 ml TEN buffer (10 mM Tris-HCl pH 8.0, 100 mM EDTA, 150 mM NaCl), centrifuged (1500× *g*, 2 min, 4 °C) and resuspended in 300 µL SDS-EB buffer (2% *w/v* SDS, 400 mM NaCl, 40 mM EDTA, 100 mM Tris-HCl pH 8.0). Extraction by mechanical destruction was carried out by treating the suspensions in Precellys Homogenizer (Bertin Instruments) with 100 mg glass beads (8 cycles, 30 s each cycle, 8000 rpm). Samples were treated with 700 µL phenol:chloroform:isoamyl alcohol (25:24:1), centrifuged (15,000× *g*, 5 min, 4 °C), then 2.5 volumes of ethanol and 0.11 volumes of Na-acetate 3M pH 5.5 were added to the supernatant. Upon incubation at −80 °C for 30 min, DNA was precipitated (15,000× *g*, 25 min, 4 °C) and washed twice with 600 µL ethanol. Pellets were resuspended in a small volume of 10 mM Tris-HCl buffer pH 8.0, treated with RNAse 10 µg/µL at 37 °C for 30 min, then DNA was further purified from RNA contaminants (NucleoSpin Plasmid, MACHEREY-NAGEL). DNA samples were quantified by Qubit 2.0 Fluorometer (Invitrogen, Carlsbad, CA, USA). Celero DNA-Seq Library Preparation Kit (Tecan Genomics, Redwood City, CA, USA) has been used for library preparation following the manufacturer’s instructions. Final libraries were checked with both Qubit 2.0 Fluorometer (Invitrogen, Carlsbad, CA, USA) and Agilent 2100 Bioanalyzer DNA assay (Agilent technologies, Santa Clara, CA, USA). Libraries were then pooled for sequencing and sequenced in paired-end 125 bp mode on HiSeq2500 (Illumina, San Diego, CA, USA). Sequencing was outsourced (https://www.igatechnology.com, accessed on 1 May 2021).

Assessment of raw read quality was performed with FASTQC v0.11.9 (https://www.bioinformatics.babraham.ac.uk/projects/fastqc/, accessed on 1 May 2021). All sequences were trimmed with Trimmomatic v0.39 [103]. Alignments were performed with Burrows–Wheeler Aligner (BWA) v0.7.17 [104] using default parameters for paired-end reads and mapped to the recently published reference genome [105]. Sorting and deduplication were performed with PicardTools v2.17.10 (http://broadinstitute.github.io/picard, accessed on 1 May 2021). Statistical analysis of BAM files was performed with QualiMap v2.2.2 [106]. SNPs and small indels were called using the GATK Haplotype Caller v4.0.6.0 [107] with ploidy set to 1. All the filtering processes were carried out using GATK *SelectVariants* and *VariantFilteration* tools. First, common SNPs between each mutant and the parental WT were removed from the dataset. The *Quality by Depth* filter with a cut-off of QD < 2.0 was used to filter out variants with low confidence. In addition, *Chlorella vulgaris* is haploid and heterozygous sites likely represent alignment or sequencing errors. Therefore, heterozygous variants in each sample with an allele frequency below 0.9 were removed. Finally, variants were manually inspected using the Integrated Genomics Viewer (IGV) v2.3 [108]. SnpEff v4.3 [109] was used to retrieve the genomic context of each variant and predict the effect of the variants on gene function. Functional information for the identified genes was obtained through Blast2go analysis [110]. Protein domains and motifs were predicted by InterPro and its associated software [111]. SIFT 4G (Sorting Intolerant From Tolerant for Genomes) algorithm v2.0.0 [112] was used to build SIFT4G database for *Chlorella vulgaris* genome. SIFT 4G annotator (https://github.com/pauline-ng/SIFT4G_Annotator, accessed on 1 May 2021) was used to annotate the VCF file. TargetP v2.0 [113] and ChloroP v1.1 [114] were used to detect sequence signals.

## Figures and Tables

**Table 1:** Pigment content, PSII maximum quantum yield (F_v_/F_m_) and PSII functional antenna size of WT and mutants *NRF*. Parameters were determined after 5 days of growth in minimal medium BG-11. Data are expressed as mean ± SD, *n* > 4. Significantly different values (ANOVA followed by Tukey’s post-hoc test at a significance level of *p* < 0.05), within the same column, are marked with different letters.

Genotype	Chl/Cell (pg)	Car/Cell (fg)	Chl *a*/*b*	F_v_/F_m_	PSII Antenna Size (T_2/3_^−1^·10^3^, ms^−1^)
**WT**	0.26 ± 0.03 ^a^	57 ± 3 ^a^	2.67 ± 0.22 ^a^	0.67 ± 0.03 ^a^	6.74 ± 0.49 ^a^
***NFR-3***	0.11 ± 0.02 ^b^	71 ± 3 ^b^	4.14 ± 0.35 ^b^	0.66 ± 0.03 ^a^	4.32 ± 0.22 ^b^
***NFR-13***	0.07 ± 0.01 ^c^	65 ± 2 ^c^	4.92 ± 0.46 ^c^	0.65 ± 0.04 ^a^	3.19 ± 0.42 ^c^

**Table 2:** HPLC analysis of pigment composition of WT and *NFR* mutants upon growth in either control or N-depleted media. Pigment composition was determined after 5 days of growth in each medium. Cells were dark-adapted for 2 h before pigment extraction in dimethyl-formamide (DMFA). Data are expressed as mean ± SD, *n* = 3, and normalized to 100 Chls. Significantly different values (ANOVA followed by Tukey’s post-hoc test at a significance level of *p* < 0.05), within the same column, are marked with different letters.

					mol/100 mol Chl						
	Genotype	Car/Cell (fg)	Chl *a*/*b*	Chl/Car	Neo	Viola	Anthera	Lute	Zea	α-Car	β-Car
Control	**WT**	57 ± 3 ^a^	2.7 ± 0.2 ^a^	4.3 ± 0.1 ^a^	2.8 ± 0.8 ^a^	2.8 ± 0.7 ^a^	0.1 ± 0.1 ^a^	13.5 ± 1.7 ^a^	1.3 ± 0.1 ^a^	0.5 ± 0.1 ^a^	1.9 ± 0.3 ^a^
	***NFR-3***	71 ± 3 ^b^	4.1 ± 0.3 ^b^	1.6 ± 0.1 ^c^	5.3 ± 1.3 ^b^	8.9 ± 1.7 ^b^	1.4 ± 0.2 ^c^	37.3 ± 8.7 ^c^	5.8 ± 1.1 ^c^	0.8 ± 0.2 ^a^	4.9 ± 1.0 ^b,c^
	***NFR-13***	65 ± 2 ^c^	4.9 ± 0.4 ^b^	1.0 ± 0.3 ^d^	4.6 ± 0.5 ^b^	15.5 ± 3.0 ^c^	2.0 ± 0.5 ^c,d^	54.9 ± 5.0 ^d^	7.9 ± 1.6 ^c^	1.5 ± 0.2 ^b^	9.5 ± 1.6 ^c,d^
N-depleted	**WT**	89 ± 4 ^d^	2.8 ± 0.1 ^a^	2.8 ± 0.1 ^e^	3.9 ± 0.5 ^b^	3.1 ± 0.4 ^a^	1.1 ± 0.2 ^c^	21.5 ± 0.3 ^b^	2.9 ± 0.3 ^b^	0.7 ± 0.1 ^a^	2.9 ± 0.1 ^b^
	***NFR-3***	121 ± 2 ^e^	4.1 ± 0.1 ^b^	0.9 ± 0.1 ^d^	14.7 ± 1.1 ^d^	18.8 ± 3.9 ^e^	3.1 ± 0.8 ^d^	52.9 ± 1.1 ^d^	11.2 ± 0.8 ^d^	1.8 ± 0.1 ^b^	8.4 ± 0.1 ^d^
	***NFR-13***	160 ± 8 ^f^	4.8 ± 0.2 ^b^	0.4 ± 0.1 ^d^	27.7 ± 7.1 ^e^	40.0 ± 0.6 ^f^	4.7 ± 1.7 ^d^	116.3 ± 2.6 ^e^	21.9 ± 0.4 ^e^	2.9 ± 0.2 ^c^	19.8 ± 0.2 ^e^

**Table 3:** Photosynthesis and respiration rates. Parameters were measured on dark-adapted cell suspensions of WT and *NFR* strains, upon 5 days of photoautotrophic growth in BG-11 medium in low light conditions (70 µmol photons m^−2^ s^−1^, 25 °C). O_2_ evolution and consumption were measured with a Clark-type oxygen electrode (Oxygraph, Hansatech UK). Data are expressed as mean ± SD, *n* > 4. Significantly different values (ANOVA followed by Tukey’s post-hoc test at a significance level of *p* < 0.05), within the same row, are marked with different letters.

Parameters.	WT	*NFR-3*	*NFR-13*
Half-saturation intensity (µmol photons m^−2^ s^−1^)	110 ± 24 ^a^	128 ± 12 ^a^	241 ± 73 ^b^
P_max_ (µmol O_2_ mg Chl^−1^ h^−1^)	96 ± 5 ^a^	225 ± 2 ^b^	323 ± 45 ^c^
Respiration (µmol O_2_ mg Chl^−1^ h^−1^)	25 ± 3 ^a^	52 ± 7 ^b^	73 ± 6 ^c^
Respiration (fmol oxygen cell^−1^ h^−1^)	6.4 ± 0.8 ^a^	5.7 ± 0.7 ^a^	5.1 ± 0.4 ^a^
P_max_/respiration (relative units)	3.9 ± 0.4 ^a^	4.4 ± 0.5 ^b^	4.4 ± 0.4 ^b^

**Table 4:** Growth parameters of WT and *NFR* strains, cultured in air/CO_2_ bubbling system. Biomass content was measured by determination of dry biomass accumulated at the end of the cultivation period, divided by the number of days of cultivation. µ, specific growth rate, was calculated from the slope of logarithmic cell concentration curve. Data are expressed as mean ± SD, *n* = 5. Significantly different values (ANOVA followed by Tukey’s post-hoc test at a significance level of *p* < 0.05) are marked with different letters.

Genotype	Mean Increase of Biomass (g L^−1^ day^−1^)	µ (day^−1^)
**WT**	0.43 ± 0.03 ^a^	1.87 ± 0.08 ^a^
***NFR-3***	0.56 ± 0.03 ^b^	1.99 ± 0.04 ^b^
***NFR-13***	0.56 ± 0.01 ^b^	1.99 ± 0.02 ^b^

**Table 5:** Summary of whole-genome sequencing and read mapping for WT and two EMS-treated mutants.

Sample	Total Number of Reads Produced	Total Number of Reads after Trimming	Mapped Reads	Mean Fold Coverage
**WT**	38,406,276	25,308,558	25,157,713	65.9×
***NFR-3***	37,378,884	28,743,282	20,555,916	55.2×
***NFR-13***	17,628,716	11,931,178	7,249,676	18.6×

**Table 6:** Identified variants that are specific for the respective mutant (SS: Super-Scaffold; PO: Pilon Object; sub: substitution; noTP: no Transit Peptide; SP: Signal Peptide; mTP: mitochondrial Transit Peptide; cTP: chloroplast Transit Peptide; * indicates low confidence for SIFT algorithm prediction—see Materials and Methods for details).

Mutant	Variant pos.	Ref/Alt		Region	CDS Effect [SIFT Prediction]	TargetP	ChloroP	Putative Gene Function (Gene ID)
*NFR-13*	SS_1:3929152	G	A	Exon	Missense sub. (Arg598Gln) [SIFT: Tolerated]	noTP	-	Predicted protein (g380)
*NFR-13*	SS_2:14375	G	A	Exon	Missense sub. (Arg150Cys) [SIFT: Deleterious]	noTP	-	RNA helicase, activating signal cointegrator 1 (g5804)
*NFR-13/NFR-3*	SS_2:5043375	G	C	Exon	Missense sub. (Gln67His) [SIFT: Tolerated]	noTP	-	Predicted protein (g2438)
*NFR-13*	SS_3:393571	G	A	Exon	Missense sub. (Arg470Cys) [SIFT: Deleterious *]	noTP	-	Serine/threonine-protein phosphatase 2A 65 kDa regulatory subunit A beta isoform-like (g2544)
*NFR-13/NFR-3*	SS_5:2569375	G	C	Exon	Missense sub. (Glu178Asp) [SIFT: Deleterious]	noTP	cTP	Glycerol-3-phosphate acyltransferase 3 (g4271)
*NFR-13*	SS_5:2624939	C	T	Exon	Missense sub. (Ser119Leu) [SIFT: Deleterious]	SP	-	Serine threonine- kinase receptor R831 (g4284)
*NFR-3*	SS_10:492953	G	A	Exon	Missense sub. (Arg233Cys) [SIFT: Deleterious]	noTP	-	S49 family peptidase (g6424)
*NFR-13*	SS_13:1005023	G	A	Exon	Missense sub. (Ala80Thr) [SIFT: Tolerated]	mTP	cTP	Acylamino-acid-releasing enzyme-like (g7937)
*NFR-3*	SS_14:552899	G	A	Exon	Missense sub. (Gly391Asp) [SIFT: Deleterious]	noTP	-	Biosynthetic arginine decarboxylase (g9015)
*NFR-13*	SS_14:1569725	C	T	Exon	Missense sub. (Ala6Thr) [SIFT: Deleterious *]	noTP	-	Hypothetical protein (g8357)
*NFR-13/NFR-3*	SS_18:1233529	G	A	Exon	Missense sub. (Gly1235Arg) [SIFT: Deleterious *]	noTP	-	Hypothetical protein (g9528)
*NFR-13/NFR-3*	SS_1:4651287	G	A	Exon	Synonymous sub.	noTP	-	cold shock domain-containing protein (g211)
*NFR-13/NFR-3*	SS_2:4256144	C	T	Exon	Synonymous sub.	noTP	-	haloalkane dehalogenase (g2220)
*NFR-13/NFR-3*	SS_2:4392343	C	G	Exon	Synonymous sub.	noTP	-	solute carrier family 25 member 44 (g2251)
*NFR-13/NFR-3*	SS_5:1173035	G	A	Exon	Synonymous sub.	noTP	-	Large subunit GTPase 1 (g3878)
*NFR-13/NFR-3*	PO_26F:57866	C	T	Exon	Synonymous sub.	noTP	-	Predicted protein (g10631)
*NFR-3*	SS _1:3944123	C	T	Intron	No	mTP	cTP	Methionyl-tRNA synthetase (g376)
*NFR-13/NFR-3*	SS_1:5342472	G	A	Intron	No	noTP	cTP	Cleavage and polyadenylation specificity factor subunit 1 (g15)
*NFR-13/NFR-3*	SS_2:4545066	G	A	Intron	No	noTP	-	Putative Xaa-Pro aminopeptidase 3 (g2293)
*NFR-13/NFR-3*	SS_2:4545075	G	A	Intron	No	noTP	-	Putative Xaa-Pro aminopeptidase 3 (g2293)
*NFR-13/NFR-3*	SS _3:1002678	A	G	Intron	No	SP	-	Hypothetical protein (g2664)
*NFR-13/NFR-3*	SS_5: 1971361	AG	A	Intron	No	noTP	-	MAU2 chromatid cohesion factor-like protein (g4126)
*NFR-3*	SS _5:2687539	G	C	Intron	No	noTP	cTP	Serine/threonine-protein kinase sepA (g4294)
*NFR-13/NFR-3*	SS_6: 1727681	TC	T	Intron	No	cTP	cTP	Water chloroplastic-like (g4763)
*NFR-13/NFR-3*	SS _13:303225	G	A	Intron	No	noTP	cTP	Allophanate hydrolase (g7748)
*NFR-3*	SS_13:1920503	G	A	Intron	No	noTP	-	U3 small nucleolar ribonucleoprotein protein IMP4 (g8749)
*NFR-3*	SS_7:737237	G	C	Intron	No	noTP	-	Sodium:proline symporter (g5015)
*NFR-3*	SS_8:332225	C	T	Intron	No	noTP	cTP	MFS general substrate transporter (g5411)
*NFR-13*	SS_1:269018	C	A	Splice region	No	noTP	-	Preprotein translocase subunit SecA (g1481)
*NFR-13/NFR-3*	SS_3:344676	G	A	Splice donor	Yes	noTP	-	E3 Ubiquitin-protein Ligase SP1 related [PTHR47568:SF2] (g2532)
*NFR-13/NFR-3*	SS_13:2646657	G	A	Splice region	No	cTP	cTP	S1 motif domain-containing protein (g8549)
*NFR-13*	SS_1:2991332	G	T	Intergenic	No	noTP	-	Upstream of: Nuclear/nucleolar GTPase 2 (g672); Nuclear transport receptor (g671)
*NFR-13/NFR-3*	SS_5:1304829	C	T	Intergenic	No	noTP	-	Upstream of predicted protein ( g3913). Downstream of predicted protein (g3914)
*NFR-3*	SS1:3529923	A	AT	Intergenic	No	noTP	-	Downstream of transcription initiation factor IIB-2 (g501). Downstream of putative phosphatase 2C 35 (g500)

## Data Availability

Not applicable.

## Supplementary Materials

The following are available online at https://www.mdpi.com/article/10.3390/plants10050911/s1. Figure S1. Analysis of pigment content of WT and *NFR* cells, grown in either control or N-depleted media. Pigment composition was determined after 5 days of growth in each medium (see Table 2). Separation of lipid-soluble pigments was based on HPLC analysis. Each chromatogram represents absorbance at 440 nm of pigments extracted in dimethyl-formamide from dark-adapted cells. Chromatograms were vertically shifted for better comparison. Figure S2. Effect of nitrogen starvation on WT and *NFR* mutants. (A) Cells were grown in BG-11 with 100% content of nitrogen (17 mM NaNO_3_) till they reach saturation. Then the cells were collected by centrifugation and then resuspended in BG-11 with 5% content of nitrogen (0.8 mM NaNO_3_). Yellow circles represent the intracellular content in lipophilic compounds, such as TAGs and carotenoids, whose accumulation is stimulated by factors such as irradiance and nutrient availability (see [75] in the main text). (B) Appearance of the three genotypes at the end of the experiment, after 5 days of growth in N-depleted medium. All the lines were diluted at the same cellular concentration (2 × 10^7^ cells mL^−1^). Figure S3. Growth of WT and *NFR* strains under low light conditions. (A) Growth curves of WT and *NRF* mutant lines under photoautotrophic conditions, monitored as the cell number per ml culture. All experiments were performed in flasks with continuous stirring, illuminated with 70 μmol photons m^−2^ s^−1^, 25 °C. Cell concentration at t_0_ was about 1 × 10^6^ cells mL^−1^. (B) Fresh weight (FW) of biomass collected after 9 days of growth. Symbols and error bars show means ± SD, *n* = 5. Values marked with different letters are significantly different from each other (ANOVA, *p* < 0.05). Figure S4. Photoautotrophic growth of WT and *NFR* strains under excess light conditions. (A) Growth of WT and *NRF* mutant lines was monitored under autotrophic conditions, in flasks with continuous stirring, under strong light conditions (2500 µmol photons m^−2^ s^−1^, 25 °C), as the cell number per ml culture. At time 0, the cultures were switched from low light (70 μmol photons m^−2^ s^−1^, 25 °C) to strong light conditions. Cell concentration at t_0_ was about 1 × 10^6^ cells mL^−1^. (B) fresh weight (FW) of biomass collected after 6 days of growth. Symbols and error bars show means ± SD, *n* = 5. Values marked with different letters are significantly different from each other (ANOVA, *p* < 0.05). Figure S5. Analysis of room temperature Chl fluorescence during photosynthesis. Chl fluorescence was monitored in cell suspensions from dark-adapted cultures (see methods for details). Cells were given 25 min of white light illumination, over a range of light intensities (white bar). Maximum fluorescence in the dark (F_m_, dark bar) and in the light (F_m_’, white bar) are shown as means ± SD (*n* = 4). Figure S6. Frequency of base changes in NFR mutants. EMS mutagenesis induces nucleotide transition, transversion, insertion and deletions. Higher frequency of G/C to A/T transition corresponds to the expected EMS mutagenesis outcome with transitions as the predominant type of mutations. Supplementary Figure S7. Schematic representation of Glycerol-3-phosphate acyltransferase 3 (g4271) and predicted domains. (A) An overview of protein domains and motifs predicted by InterPro and its associated software. (B) The last two residues of the cytoplasmic domain are shaded light red (glutamic acid 178 and arginine 179). The site of mutation E178D overlaps with PANTHER entry PTHR23063:SF2, SUPERFAMILY entry SSF69593, and predicted cytoplasmic domain. (C) Amino acid sequence alignment of predicted cytoplasmic domain (2) and transmembrane domain (3) in *Chlorella vulgaris* Glycerol-3-phosphate acyltransferase 3 (g4271) and top BLAST hits in green algae (taxid:3041) with ≥75% coverage. The position of Glu178Asp mutation present in both *NFR-13* and *NFR-3* mutants is indicated by an inverted red triangle. The cytoplasmic domain in *Chlorella vulgaris* is indicated by teal color while the transmembrane domain is in orange. Black boxes indicate ≥80% sequence identity. Supplementary Table S1. Lipid content of algal biomass. Total lipid content was determined gravimetrically on the dry biomass, from WT and mutant cultures grown for 7 days in nutrient-rich BG-11 medium (1400 μmol photons m^−2^ s^−1^, 25 °C) and then moved for further 4 days of growth in modified BG-11 medium with limiting N source. Data are expressed as mean ± SD, *n* = 4. Significant different values in oil content among genotypes (ANOVA test, *p* < 0.05) are marked with different letters.
