# Supplementary material for: High Carotenoid Mutants of Chlorella vulgaris Show Enhanced Biomass Yield under High Irradiance
Source: Plants (Basel). 2021 May 1;10(5):911. doi: 10.3390/plants10050911 (PMC8147269; doi:10.3390/plants10050911)
Supplement: Supplementary file 1 [file plants-10-00911-s001.zip › plants-1192129-supplementary.pdf]

plants

Supplementary Results

Supplementary Figure S1

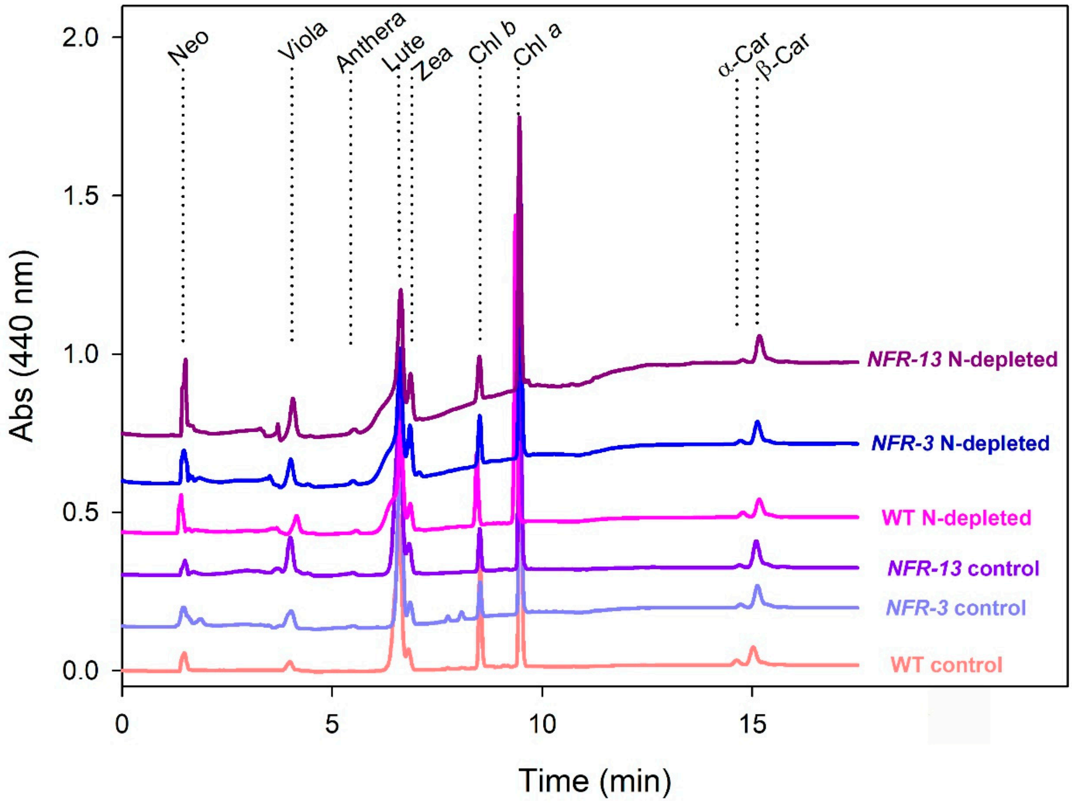

Figure S1. Analysis of pigment content of WT and *NFR* cells, grown in either control or N-depleted media. Pigment composition was determined after 5 days of growth in each medium (see Table 2). Separation of lipid-soluble pigments was based on HPLC analysis. Each chromatogram represents absorbance at 440 nm of pigments extracted in dimethylformamide from dark-adapted cells. Chromatograms were vertically shifted for better comparison.

Supplementary Figure S2

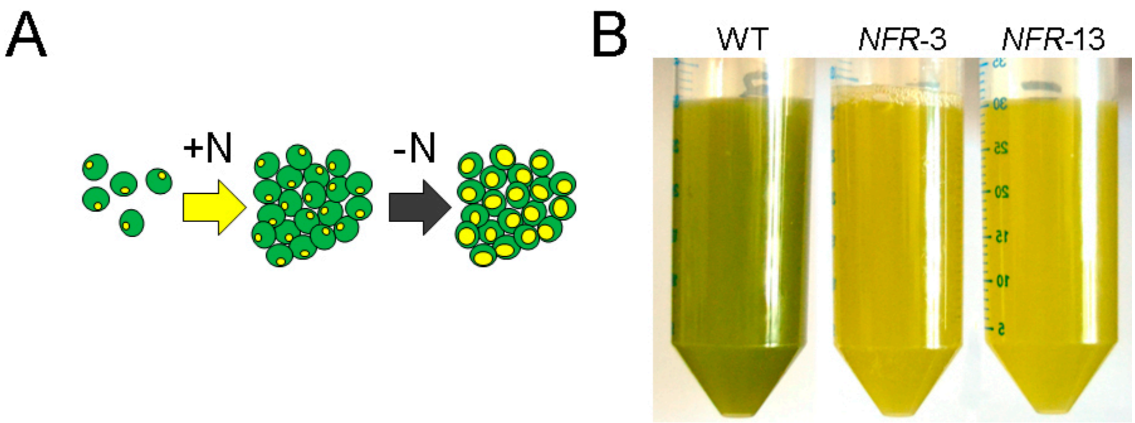

Figure S2. Effect of nitrogen starvation on WT and *NFR* mutants. (A) Cells where grown in BG-11 with 100% content of nitrogen (17 mM $NaNO_3$) till they reached saturation. Then the cells where collected by centrifugation and resuspended in BG-11 with 5% content of nitrogen (0.8 mM $NaNO_3$). Yellow circles represent the intracellular content in lipophilic compounds, such as TAGs and carotenoids, whose accumulation is stimulated by factors such as irradiance and nutrient availability (see reference [74] in the main text). (B) Appearance of the three genotypes at the end of the experiment, after 5 days of growth in N-depleted medium. All lines were diluted at the same cellular concentration ($2 \cdot 10^7$ cells ml^{-1}).

Supplementary Figure S3

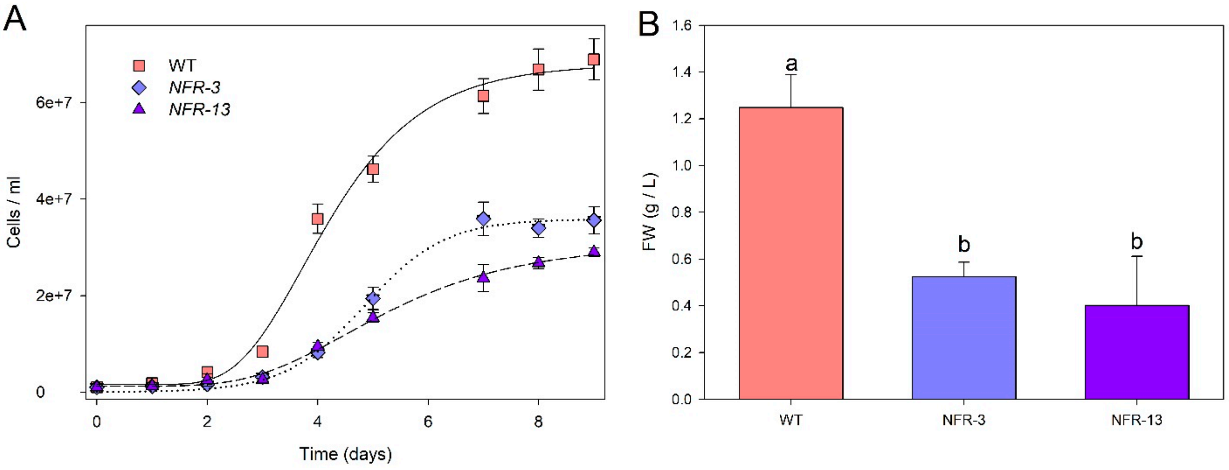

Figure S3. Growth of WT and *NFR* strains under low light conditions. (A) Growth curves of WT and *NRF* mutant lines under photoautotrophic conditions, monitored as the cell number per ml culture. All experiments were performed in flasks with continuous stirring, illuminated with 70 µmol photons m^{-2} s^{-1}, 25 °C. Cell concentration at t_0 was about $1 \cdot 10^6$ cells mL^{-1}. (B) Fresh weight (FW) of biomass collected after 9 days of growth. Symbols and error bars show means ± SD, n = 5. Values marked with different letters are significantly different from each other (ANOVA followed by Tukey's post-hoc test at a significance level of $P < 0.05$).

Supplementary Figure S4

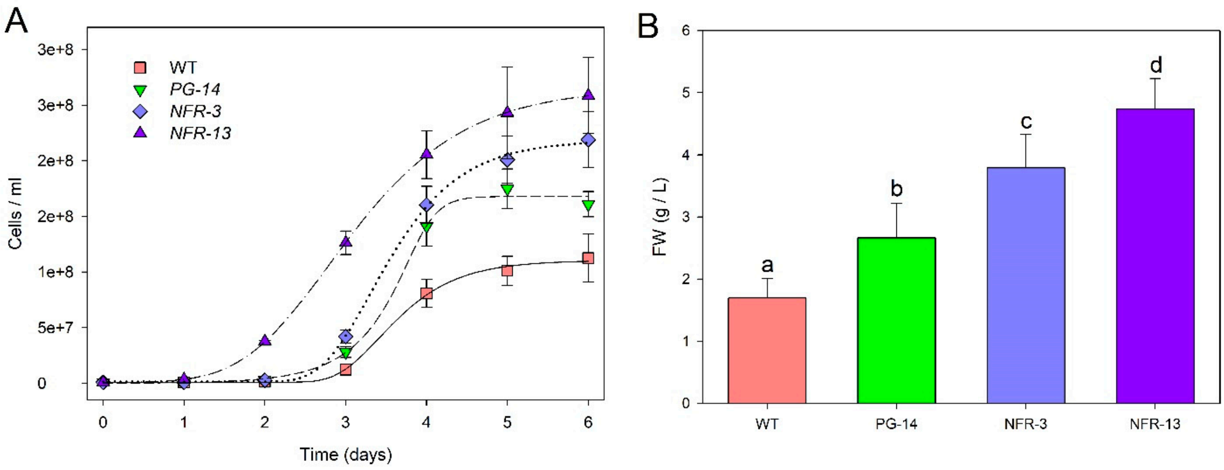

Figure S4. Photoautotrophic growth of WT and *NFR* strains under excess light conditions. (A) Growth of WT and *NRF* mutant lines was monitored under autotrophic conditions, in flasks with continuous stirring, under strong light conditions (2,500 µmol photons m^{-2} s^{-1}, 25°C), as the cell number per ml culture. At time 0, the cultures were switched from low light (70 µmol photons m^{-2} s^{-1}, 25°C) to strong light conditions. Cell concentration at t_0 was about $1{\cdot}10^6$ cells mL^{-1}. (B) Fresh weight (FW) of biomass collected after 6 days of growth. Symbols and error bars show means ± SD, n = 5. Values marked with different letters are significantly different from each other (ANOVA followed by Tukey's post-hoc test at a significance level of $P < 0.05$).

Supplementary Figure S5

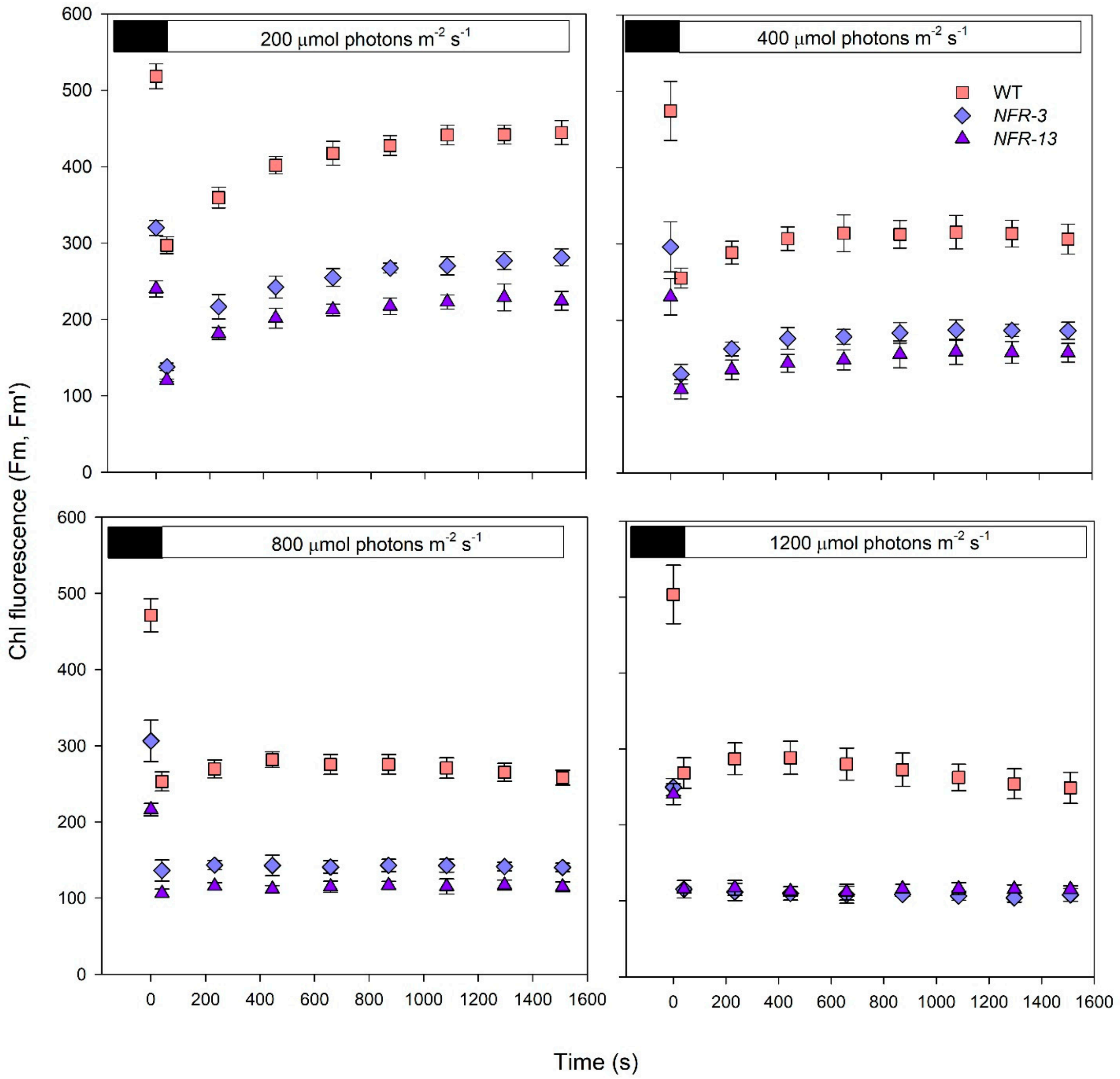

Figure S5. Analysis of room temperature Chl fluorescence during photosynthesis. Chl fluorescence was monitored in cell suspensions from dark-adapted cultures (see methods for details). Cells were given 25 min of white light illumination, over a range of light intensities (white bar). Maximum fluorescence in the dark (F_m, dark bar) and in the light (F_m', white bar) are shown as means ± SD (n = 4).

Supplementary Figure S6

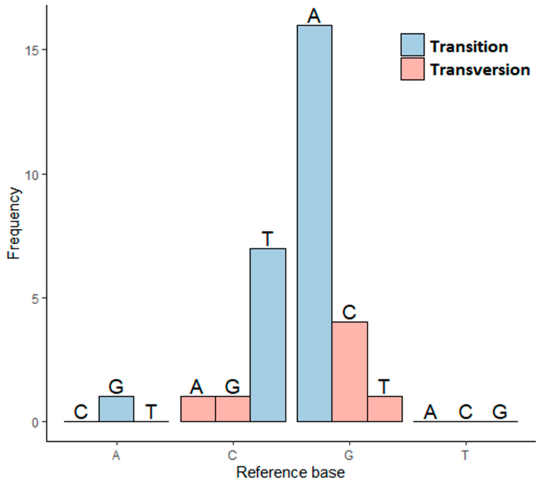

Figure S6. Frequency of base changes in *NFR* mutants. EMS mutagenesis induces nucleotide transition, transversion, insertion and deletions. Higher frequency of G/C to A/T transition corresponds to the expected EMS mutagenesis outcome with transitions as the predominant type of mutations.

Supplementary Figure S7

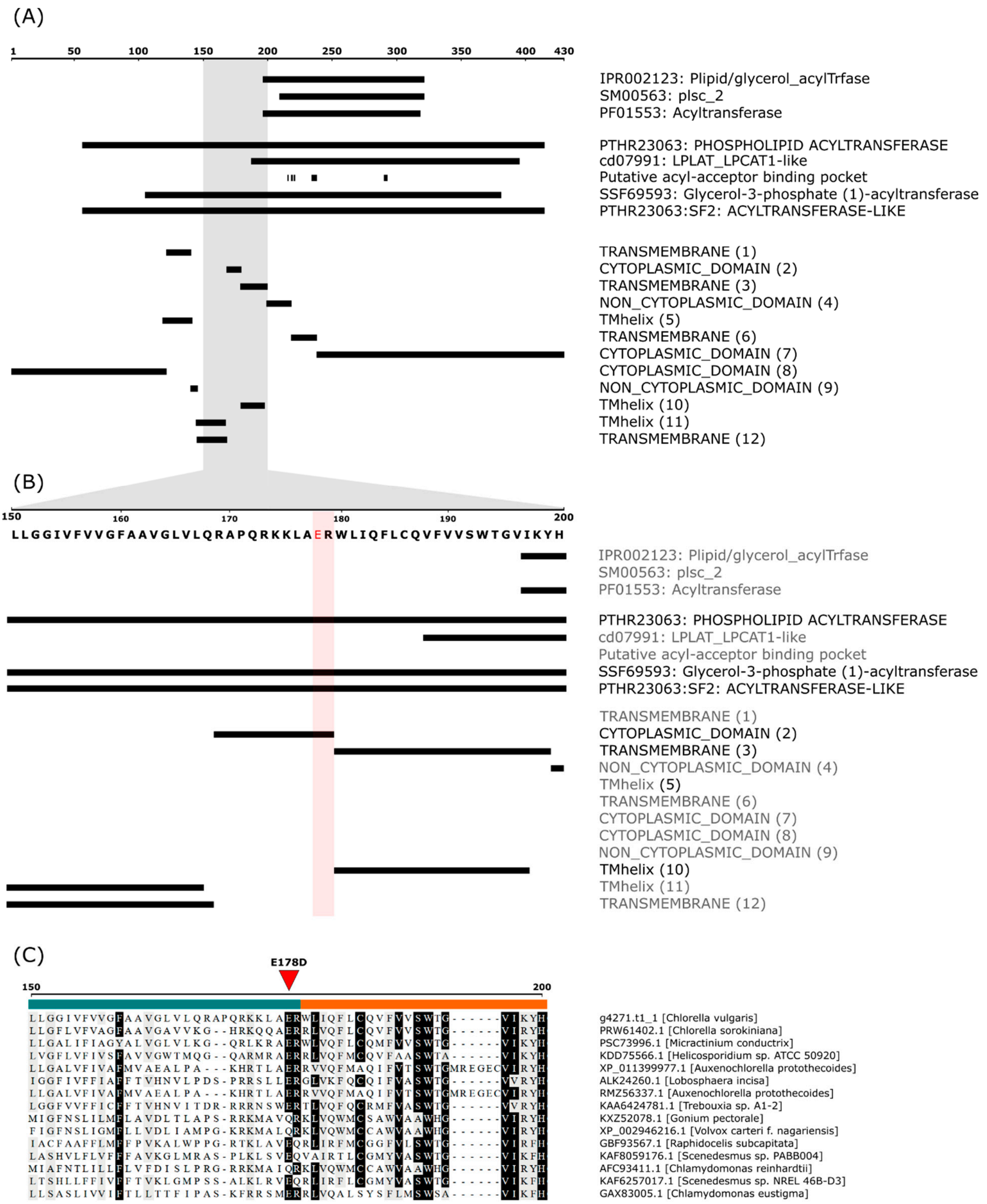

Figure S7. Schematic representation of Glycerol-3-phosphate acyltransferase 3 (g4271) and predicted domains. (A) An overview of protein domains and motifs predicted by InterPro and its associated software. (B) The last two residues of the cytoplasmic domain are shaded light red (glutamic acid 178 and arginine 179). The site of mutation E178D

overlaps with PANTHER entry PTHR23063:SF2, SUPERFAMILY entry SSF69593, and predicted cytoplasmic domain. (C) Amino acid sequence alignment of predicted cytoplasmic domain (2) and transmembrane domain (3) in *Chlorella vulgaris* Glycerol-3-phosphate acyltransferase 3 (g4271) and top BLAST hits in green algae (taxid:3041) with ≥75% coverage. The position of Glu178Asp mutation present in both *NFR-13* and *NFR-3* mutants is indicated by an inverted red triangle. The cytoplasmic domain in *Chlorella vulgaris* is indicated by teal color while the transmembrane domain is in orange. Black boxes indicate ≥80% sequence identity.

Supplementary Table S1

genotype	Total oil content (% DW)
**WT**	25.1 ± 2.7[a]
NFR-3	16.5 ± 3.0[b]
NFR-13	15.7 ± 1.1[b]

Supplementary Table S1. Lipid content of algal biomass. Total lipid content was determined gravimetrically on the dry biomass, from WT and mutant cultures grown for 7 days in nutrient-rich BG-11 medium (1,400 μmol photons m^{-2} s^{-1}, 25 °C) and then moved for further 4 days of growth in modified BG-11 medium with limiting N source. Data are expressed as mean ± SD, $n = 4$. Significant different values in oil content among genotypes (ANOVA followed by Tukey's post-hoc test at a significance level of $P < 0.05$) are marked with different letters.
